# Electrical dry needling versus conventional physiotherapy in the treatment of active and latent myofascial trigger points in patients with nonspecific chronic low back pain

**DOI:** 10.1186/s13063-022-06179-y

**Published:** 2022-03-28

**Authors:** Inmaculada Carmen Lara-Palomo, Esther Gil-Martínez, Eduardo Antequera-Soler, Adelaida María Castro-Sánchez, Manuel Fernández-Sánchez, Héctor García-López

**Affiliations:** Department of Nursing, Physical Therapy and Medicine, University of Almeria, Ctra. Sacramento s/n La Cañada de San Urbano, 04120 Almeria, Spain

**Keywords:** Chronic low back pain, Conventional physiotherapy, Electrical dry needling

## Abstract

**Background:**

Chronic low back pain is considered to be one of the main causes of absenteeism from work and primary and specialized consultations. The symptoms of nonspecific chronic low back pain may be accompanied by the activation of myofascial trigger points in the muscles, together with local and/or referred pain. Electrical dry needling is increasingly used in the treatment of lumbar myofascial pain. Conventional physiotherapy, however, is a popular approach to chronic pathologies, and there is evidence of different modalities of physiotherapy being used in the treatment of chronic low back pain. The aim of this study has been to determine the effectiveness of electrical dry needling versus conventional physiotherapy when applied to active and latent myofascial trigger points in patients with nonspecific chronic low back pain.

**Methods:**

This is a controlled, randomized, two-arm, double-blind study. A total of 92 patients with chronic low back pain (time to onset ≥ 3 months, Roland Morris Disability Questionnaire score ≥ 4) will be recruited from the University of Almería. Participants will be divided into two study groups (*n* = 40) to receive treatment of low back pain with electrical dry needling and conventional physiotherapy (ischaemic compression, analytic stretching and postural education training dossier). A total of 6 sessions will be administered once a week for 6 weeks. Pain intensity, disability, fear of movement, quality of life, quality of sleep, anxiety and depression, pressure pain threshold, abdominal strength and lumbar mobility will be recorded at 6 weeks (post-immediate) and 2 months after the end of treatment.

**Discussion:**

We believe that an approach including electrical dry needling to chronic low back pain dysfunction will be more effective in these patients. The results of this study will inform clinicians on which type of treatment is more beneficial for patients with chronic low back pain.

**Trial registration:**

ClinicalTrials.gov NCT04804228. Registered on 14 January 2021

**Supplementary Information:**

The online version contains supplementary material available at 10.1186/s13063-022-06179-y.

## Background

Low back pain is a major public health problem in industrialized countries that causes individual suffering, absenteeism from work and, in some cases, early retirement. Since it is a common pathology that is difficult to treat effectively, low back pain represents a high economic burden for both society and the health system [1–3] and is considered one of the main causes of primary and specialized care consultations [4]. In Europe, the direct and indirect costs of low back pain account for between 1.7 and 2.1% of the annual gross domestic product [8, 9].

Back pain, which affects up to 23% of the population worldwide, is the most common chronic disease in people under 65 years of age. With a lifetime prevalence of up to 85% in industrialized countries [3], estimates suggest that between 24 and 80% of patients have at least one recurrence per year, being more frequent and persistent in older adults [2, 5, 6]. In any given 6-month period, 72% of adults in the general population will report low back pain, and 11% will report disabling low back pain [7].

Pain intensity, degree of pain interference with activities of daily living (resulting in disability), and health-related quality of life are among the primary outcomes in studies in patients with low back pain [1]. In the 2010 Global Burden of Disease Study, which includes 291 diseases, low back pain ranked first in terms of disability and sixth in terms of overall burden [10]. In addition to age, psychological factors such as emotional distress and dysfunctional pain coping mechanisms play an important role in the development and/or persistence of nonspecific chronic low back pain (CLBP) [6].

The symptoms of CLBP may be accompanied by the activation of myofascial trigger points (MTrPs) in the lumbar and proximal muscles, together with local and/or referred pain [11, 12]. Clinically, MTrPs present as palpably taut bands with a local twitch response and pain on pressure [12–14]. When the points are active, digital palpation causes pain to radiate to a distant site (referred pain); when they are latent, palpation may be locally painful, but no radiation occurs (local pain) [11, 12].

The MTrPs of each muscle have their own characteristic pain pattern; therefore, the spread of the pain can help identify the muscles that may contain active and latent trigger points [15]. CLBP is associated with the presence of MTrP in the quadratus lumborum muscle, and often also in the lumbar and superficial paraspinal multifidus muscles [13].

Noninvasive treatment options for CLBP remain controversial, and there is no general consensus on the best approach [16]. Some trials in CLBP and electrical dry needling conclude that there is still no strong evidence to support the clinical effectiveness of electrical dry needling on LBP versus any other treatment modality [17–20].

Dry needling is typically used to treat soft tissues, such as muscles, ligaments, tendons, fascia, scar tissue, peripheral nerves and neurovascular bundles involved in a variety of neuromusculoskeletal pain syndromes [21, 22]. Dry needling involves the insertion of fine monofilament needles without the use of injectables, and its therapeutic effect is based on stimulating specific reactions in the target tissue [23–27]. It is a relatively new treatment modality used by physical therapists around the world as part of the complex treatment of chronic musculoskeletal pain [23]. The effectiveness of this approach has been confirmed in numerous studies and systematic reviews on the management of chronic lumbar MTrPs and myofascial pain [28–30]. In electrical dry needling, needle electrodes are used to deliver an electric current to the taut muscle band or the pain-generating MTrP [25, 26, 31] Low-frequency currents are thought to improve the physiological effects of the therapy by using electrical stimulation to enhance certain physiological reactions and achieve a speedier analgesic and anaesthetic effect than that obtained with standard dry needling in patients with low back pain [32, 33]. Despite the popularity of electrical dry needling in clinical physiotherapy, there is insufficient scientific evidence to show its therapeutic effects in the treatment of CLBP [16, 34, 35].

Various treatment approaches beyond the scope of physiotherapy have been proposed to reduce the recurrence of low back pain and its associated care costs. Clinical practice guidelines provide strong evidence that cognitive behavioural therapy, exercise, spinal manipulation and rehabilitation with various physiotherapy procedures are all moderately effective in chronic or subacute low back pain (> 4 weeks duration) [34–36]. Recent systematic reviews and meta-analyses recommend exercise therapy to improve back strength, flexibility, range of motion and fitness in chronic low back pain [37–39]. However, there is no evidence to show whether invasive approach like the electrical dry needling is more effective than a conventional physiotherapy in patients with nonspecific CLBP.

## Study objectives

The objective of this randomized controlled trial is to evaluate the effectiveness of electrical dry needling versus conventional physiotherapy in the treatment of patients with nonspecific chronic lower back pain.

The specific objectives are (i) to compare the effectiveness of electrical dry needling versus conventional physiotherapy in improving pain, functionality, lumbar spine mobility and quality of life in patients with nonspecific chronic low back pain and (ii) to evaluate the effect of this therapy on active myofascial trigger points in terms of the pressure tolerance threshold following electrical dry needling versus conventional physiotherapy.

## Methodology

### Study design and ethical approval

This is a controlled, randomized, two-arm, double-blind study comparing (i) patients with chronic low back pain treated with electrical dry needling and (ii) patients with chronic low back pain treated with conventional physiotherapy consisting of ischaemic compression, analytical stretching and a dossier of home lumbar spine exercises. Study participants will be randomly assigned to two groups (electrical dry needling group or conventional physiotherapy group) with a 1: 1 ratio.

This protocol has been drawn up following the Standard Protocol Items: Recommendations for Interventional Trials (SPIRIT) (Additional file 4). The study will be carried out in partnership with the physiotherapy department of the University of Almería. Ethical approval for this trial was granted by the University of Almería Research Ethics Committee (UALBIO2020/044). The study protocol was registered in an international clinical trial registry, ClinicalTrials.gov (protocol number NCT04804228).

### Participants

**Fig. 1:**
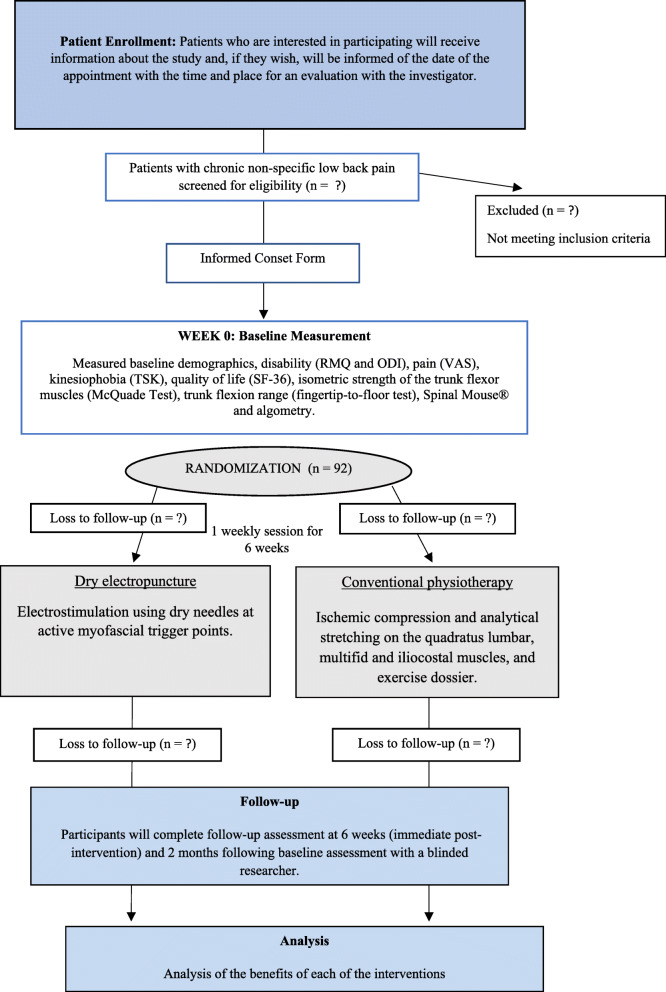
Design and flow of participants through the trial. RMQ, Roland-Morris Low Back and Disability Questionnaire; ODI, Oswestry Disability Index; VAS, Visual Analogue Scale; TSK, Tampa Scale for Kinesiophobia

A total of 92 patients aged between 30 and 65 years, diagnosed with nonspecific chronic low back pain lasting more than 3 months [40] who are not currently undergoing any type of treatment, will be recruited. Patients will be randomized to two treatment groups (electrical dry needling or conventional physiotherapy). Participants will receive treatment once a week for 6 weeks in the physiotherapy laboratories of the University of Almería, with a follow-up evaluation at 6 weeks and 2 months after the start of treatment. During their first visit, participants will be screened for study eligibility according to the study inclusion and exclusion criteria and will be assessed by a therapist blinded to the interventions. After this face-to-face evaluation, patients will be randomly assigned to one of the two groups and will receive the corresponding treatment for low back pain administered by two researchers trained in the techniques used. All participants will sign the informed consent form, which complies with the Declaration of Helsinki of the World Health Organization (schedule of enrolment, interventions and assessments is shown in Fig. 1).

#### Inclusion criteria

Both male and female patients aged between 30 and 65 years with chronic low back pain lasting 3 months or more, with a low back pain disability score ≥ 4 on the Roland-Morris Disability Questionnaire (RMQ) and not receiving any other physiotherapy treatment are eligible for inclusion.

#### Exclusion criteria

Patients with sensory and/or coagulation disorders, a history of spinal surgery, heart complications, concurrent severe central or peripheral nervous system disease, epilepsy, needle phobia, serious pathologies that can be the main cause of chronic low back pain (for example, presence of lumbar stenosis, spondylolisthesis, tumours, etc.), or patients contraindicated for transcutaneous electrical stimulation (TENS) will be excluded.

### Randomization and blinding

Participants will be randomized to two groups using a computer-generated (Epidat 4.2) table of random numbers generated. After randomization, participants will be assigned to either the experimental electrical dry needling group or the conventional physiotherapy control group in a ratio of 1:1. Randomization will be performed by the principal investigator.

There will be 46 participants in each group. The randomly generated group allocations will be placed in sealed opaque envelopes before being delivered to the participants and stored in locked cabinets.

The outcome assessor and study statistician will be blinded to the entire process. The outcome assessor will make no attempt to guess the participant’s treatment group. The computer-generated outcome measures transmitted to the statistician will not contain any information that identifies the patient’s group.

### Interventions

After the initial evaluation, 92 patients with CLBP will be randomly assigned to one of the two groups and will receive electrical dry needling (experimental group) or conventional physiotherapy (control group). All participants will receive 1 session per week for 6 weeks, until they have received a total of 6 sessions. Patients must complete 100% of their scheduled face-to-face treatment sessions, and those in the control group must also complete 80% of the home exercise sessions in order to remain in the intention-to-treat analysis.

During the study, participants can only receive their assigned treatment; they cannot combine the study treatment with medications or any other treatment. Any interference in the treatment will be grounds for exclusion. Patients may abandon the study at any time, and the assigned interventions may be suspended or modified in a particular trial patient in response to improvement or deterioration (adverse effects) of low back pain. Adverse events will be reported to the principal investigator, who will monitor the affected patients and the possible causes of these events.

### Electrical dry needling group

**Fig. 2:**
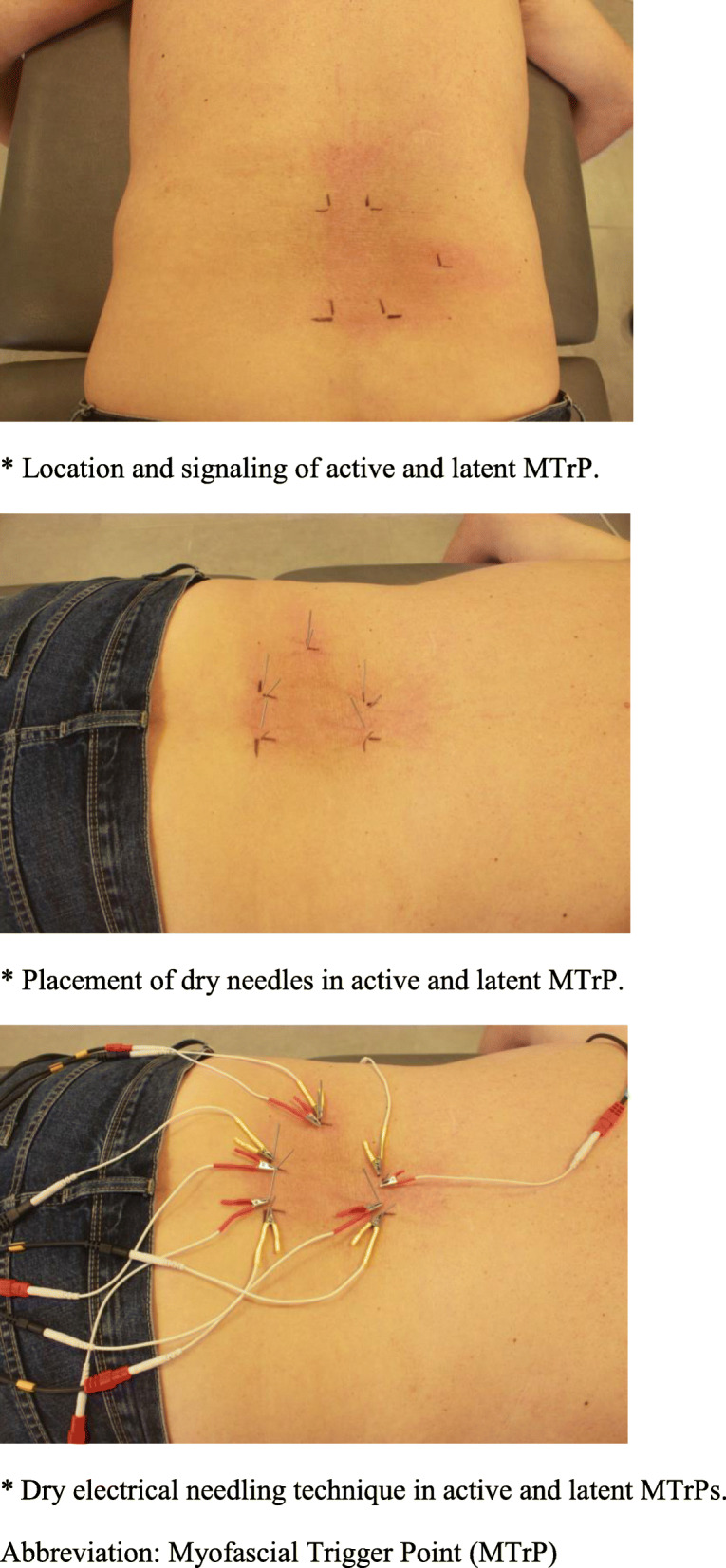
Electrical dry needling group. Location and signalling of active and latent MTrP. Placement of dry needles in active and latent MTrP. Dry electrical needling technique in active and latent MTrPs. MTrP, myofascial trigger point

Patients assigned to the electrical dry needling group (*n* = 46) will receive up to three 30-min treatment sessions (1 session per week for 6 weeks). Electrical stimulation will be applied bilaterally to the active and latent myofascial trigger points of the following muscles, following the MTrPs maps described by Travell and Simons: [2, 14] quadratus lumborum, multifidus and iliocostalis. The number of needle insertion sites will vary in each patient; the treating therapist will determine the points to be treated in each session based on whether they are active, latent or absent. Prior to needle insertion, the site will be sterilized with 70% alcohol using a cotton swab (Fig. 2).

Two sizes of sterilized disposable stainless steel acupuncture needles will be used: 0.25 mm × 30 mm or 0.30 mm × 40 mm. The size of the needle will depend on the patient’s physical constitution (i.e. muscle and/or connective tissue thickness). The needle will be inserted until it reaches the active or latent MTrP or taut band that causes the local twitch response [41]. The needles will then be connected to an electric current and left in situ for 30 min (TensMed S82-Enraf Nonius) [42, 43]. A low-frequency current (2 Hz) will be generated by a TENS device with a moderate pulse duration (250 μs) and a continuous biphasic waveform at an intensity described by the patient as “mild to moderate” [44].

### Conventional physiotherapy group

Patients assigned to the conventional physiotherapy group (*n* = 46) will receive ischaemic compression and analytical stretching of the quadratus lumborum, multifidus and iliocostal muscles once a week for a total of 6 weeks.

Ischaemic compression will consist of constant pressure stimulation with the thumb on each MTrP for between 30 s and 2 min. This compression sequence will be repeated several times. The intensity of the pressure will be adjusted to a level at which each subject reports “comfortable pain”, in order words, between the pain threshold and the maximum tolerable pain [36, 45] (see Additional file 1 for the analytical stretching procedure).

These patients will also be given a dossier of home lumbar spine exercises to be performed 5 days a week for a total of 6 weeks (Additional file 2: dossier of home exercises). To monitor compliance, patients will be instructed to note down in a booklet the dates on which they complete the exercises in the dossier.

### Data collection

At the beginning of the study, the following demographic data will be collected: age, sex, weight, height, education and clinical presentation. Primary and secondary outcome measures will be evaluated at baseline prior to randomization to different groups. This will be followed by an immediate post-treatment assessment (1 day after the final intervention) and an evaluation 2 months after the end of the intervention (short-term follow-up).

#### Primary outcome measures

The following are the primary outcome measures:

- Roland Morris Disability Questionnaire (RMDQ): This self-reported questionnaire consists of 24 items that rate limitations in different activities of daily life attributed to low back pain, such as walking, bending over, sitting, lying down, dressing, sleeping, personal care and daily activities. Disability is rated from 0 points (best) to 24 points (worst) [46].
- Oswestry Disability Index (ODI): The Oswestry Disability Index assesses the limitations in activities of daily living in 10 dimensions, each rated on a 6-point scale (0–5 points). The higher the score, the greater the disability. The overall score is expressed as a percentage and is used to classify people as minimally disabled (0–10%), moderately disabled (20–40%), severely disabled (40–60%), crippling back pain (60–80%) or bedridden (80–100%) [47].
- Visual analogue scale (VAS): Study participants will indicate the intensity of their pain on a 100-mm VAS. They are asked to situate their pain on a 100-mm horizontal line, where 0 mm indicates “no pain”, and 100 mm indicates “the worst pain imaginable” [48].

#### Secondary outcome measures

The following are the secondary outcome measures:

- Quality of life using the SF-36 Questionnaire: The SF-36 is a short-form, multipurpose health survey with only 36 questions. The instrument contains eight subscales (physical function, physical role, body pain, general health, vitality, social function, emotional role and mental health) and two summary scores: physical and mental health. Scores range from 0 to 100% and indicate the self-perceived health-related quality of life [49, 50].
- Tampa Scale for Kinesiophobia (TSK): This is a 17-item questionnaire that measures fear of movement and (re) injury. Patients rate their beliefs about their kinesiophobia on a 4-point scale ranging from strongly disagree to strongly agree [51, 52].
- Pittsburgh Sleep Quality Index (PSQI): This is a 10-item questionnaire with a total of 19 questions related to sleep habits in the previous month. The questions are divided into 7 areas, each with a score of between 0 and 3 points. The overall score ranges from 0 (no difficulty sleeping) to 21 points (severe difficulty sleeping) [53].
- Hospital Anxiety and Depression Scale (HADS): This scale consists of 14 items related to emotional distress (anxiety, depression) in populations suffering from a physical illness. It consists of two subscales (HADA: anxiety and HADD: depression) with seven items each that score from 0 (normal) to 3 (abnormal) [54, 55].
- McQuade Test: This test evaluates the isometric resistance of the flexor muscles of the trunk. The patient is placed supine and asked to flex the head and shoulders until the scapula is lifted off the table. The number of seconds they hold that position is recorded [56].
- Anterior trunk flexion. Standing, with legs straight, the patient is asked to bend forward and attempt to touch the ground. They are told to stop when pain or limitation of movement appear. The distance, in centimetres, between the fingers and the ground is measured [57].
- Spinal Mouse®: This is a safe, practical and easy-to-use instrument to measure the curvature of the spine in the frontal and sagittal planes and to assess the segmental mobility of the lumbar region [58].
- Pressure algometry (Wagner FPI 10 Algometer) in MTrPs: The algometer consists of a rubber tip and a dial that measures the pressure applied to the MTrP in increments of 0.5 kg. The pressure pain threshold will be assessed following the illustrations published by Travell and Simons [14].

### Sample size

The sample size was calculated according to the specifications established by Willian [59]. The calculations were based on the detection of differences of 2.5 points in the RMDQ (minimum detectable difference between means for a variance of 10 points in patients with chronic low back pain), assuming a standard deviation of 2.5 points, a 2-tailed test, an alpha (*α*) of 0.05 and a target power (beta) of 85%.

The following specifications will be considered: *α* = 0.05, statistical power of 85% and loss to follow-up of 15%. The sample size calculation yielded a total of 92 participants to be randomized to two intervention groups.

### Data analysis

Statistical analysis will be performed using SPSS© version 21.0 and STATA 14 using the principles of intention to treat. Comparisons will be made between the two study arms. We will calculate the difference between the groups after the final treatment session and at 2 months post-intervention (short-term results).

The efficacy variable for this clinical trial is the difference between continuous variables (i.e., RMDQ, ODI, VAS, TSK, SF-36, PSQI, HADS, pressure algometry, McQuade test and trunk range of movement) at baseline and at predetermined time points (electric dry needling treatment vs conventional physiotherapy):

The Kolmogorov-Smirnov test will be used to assess the normality of continuous variables.

The equality of means of the intragroup hypotheses will be analysed using Student’s *t* test for paired clinical variables in the case of parametric distributions and the Kruskal-Wallis *H* test in the case of nonparametric distributions.

One-way analysis of variance (ANOVA) will be used to test the intragroup hypothesis in the case of parametric distributions, and the Kruskal-Wallis *H* test will be used in nonparametric distributions.

Post hoc analysis will be obtained for parametric distributions and Mann-Whitney *U* for nonparametric distributions.

The confidence interval will be set at 95% and the level of significance at 0.05.

### Adverse effects

No potential risks have been described so far, given that these can be prevented by the operator’s knowledge of anatomy, training and experience [60]. Researchers will notify study participants of possible adverse events in the informed consent and record any adverse events that occur over the course of the study. If such events are observed, the frequency of occurrence will be analysed between the groups, and if patients have any questions or require additional information about any symptoms, they will be able to contact the physiotherapists by phone or email. Periodic reviews of security protocols will be carried out with staff.

### Ethics and dissemination

All participants will receive verbal and written information about the study before giving their consent to participate. They will be informed that they can leave the study at any time. Participants who agree to take part in the study will sign two copies of the informed consent form, one for the research and evaluation team and one for the participant.

All hard copies will be confidential and stored in a locked filing cabinet in the research group office and in electronic format in a password-protected database. The research team will monitor the integrity of the trial data. All participants, group assignments, treatment records and sociodemographic data will be coded, and the results of the questionnaires will be scored.

The data collected on each participant will be kept under lock and key by the evaluator. If the data are in digital format, they will be stored in a computer with a secret access code known only to the evaluator.

The eligibility criteria, results and analyses will not be modified once the first participant has been enrolled in the study. Any amendment to the protocol, including changes in the eligibility criteria, the results or the analyses, will be communicated to the Institutional Research Committee of the University of Almería and reported in articles and presentations disseminating the results of the trial.

The feasibility results will be published in peer-reviewed journals and presented at academic, clinical and health services conferences.

## Discussion/conclusions

Although physiotherapy with dry needling and electrical dry needling has proven positive effects on chronic low back pain [22–31], the results of studies into the duration of the analgesic effect and the dose required, for example, are contradictory [61, 62]. Therefore, further research is required to evaluate the specific components of the treatments administered by physical therapists.

This study can contribute to our understanding of the effectiveness of electrical dry needling versus conventional physiotherapy in patients with nonspecific chronic low back pain at short term. The results can help physiotherapists understand whether low back pain treated with electrical dry needling can significantly reduce disability and absenteeism due to chronic low back pain. Improving chronic low back pain without absenteeism will reduce labour costs and waiting lists for rehabilitative physiotherapy.

Due to the growing prevalence of chronic conditions such as low back pain and their impact on individuals, their circumstances and society in general, it is becoming increasingly important to provide evidence-based, cost-effective interventions [63, 64]. These interventions must first be designed, adapted and tested to determine their feasibility and cost before being evaluated in a high-quality effectiveness trial. The trial design will be reviewed based on the findings of this study before performing a definitive trial.

### Timeline

Patients will be recruited between May 2021 and August 2021. The study is expected to be completed in November 2021. Data analysis, writing of the scientific manuscript and submission to peer-reviewed scientific journals will take place from January 2022. A summary of the study outline is shown in Table 1.

**Table 1:** Time point of each assessment index

Time point	Study period						
	Enrolment		Active treatment (post-allocation)			Follow-up	
	0 W	1W	2W	4W	6W	2M	
	May to August 2021		September 2021			November 2021	
**Screening and enrolment**							
Eligibility screen RPTs	✓						
Informed consent	✓						
Eligibility screen face to face to blinded evaluator		✓					
Allocation principal investigator		✓					
**Interventions**							
Electrical dry needling (experimental group)			1 times per week				
Conventional physical therapy (control group)			1 times per week				
**Assessments**							
**Demographic variables:** Age, gender, education, occupational and marital status		✓					
**Clinical presentation of TrPs:** Location Interrogation Worsens Improvement		✓				✓	✓
**Clinical variables:** RMDQ ODI SF-36 VAS TSK PSQI HADS McQuade Test Fingertip-to-floor Spinal Mouse® Algometry		✓				✓	✓

## Supplementary Information

**Additional file 1:.** Analytical stretching exercise protocol (lumbar segment).

**Additional file 2:.** Dossier of exercises for low back pain (home program).

**Additional file 3:.** Informed Consent. Information sheet for participants.

**Additional file 4:.** Reporting checklist for protocol of a clinical trial.

## Data Availability

Not applicable.

## Abbreviations

LBP: Low back pain
MTrPs: Myofascial trigger points
ODI: Oswestry Disability Index
RMDQ: Roland-Morris Low Back and Disability Questionnaire
SF-36: Short-format health survey questionnaire
PSQI: Pittsburgh Sleep Quality Scale
HADS: Hospital Anxiety and Depression Scale
SPSS: Statistical Software Package for the Social Sciences
TENS: Transcutaneous electrical nerve stimulation
TSK: Tampa Scale of Kinesiophobia
VAS: Visual analogue scale
