# Supplementary material for: Electrical dry needling versus conventional physiotherapy in the treatment of active and latent myofascial trigger points in patients with nonspecific chronic low back pain
Source: Trials. 2022 Mar 28;23:238. doi: 10.1186/s13063-022-06179-y (PMC8961901; doi:10.1186/s13063-022-06179-y)

**APPENDIX 1. ANALYTICAL STRETCHING EXERCISE PROTOCOL (LUMBAR SEGMENT).**

1. **Stretching of the iliocostalis muscle.**


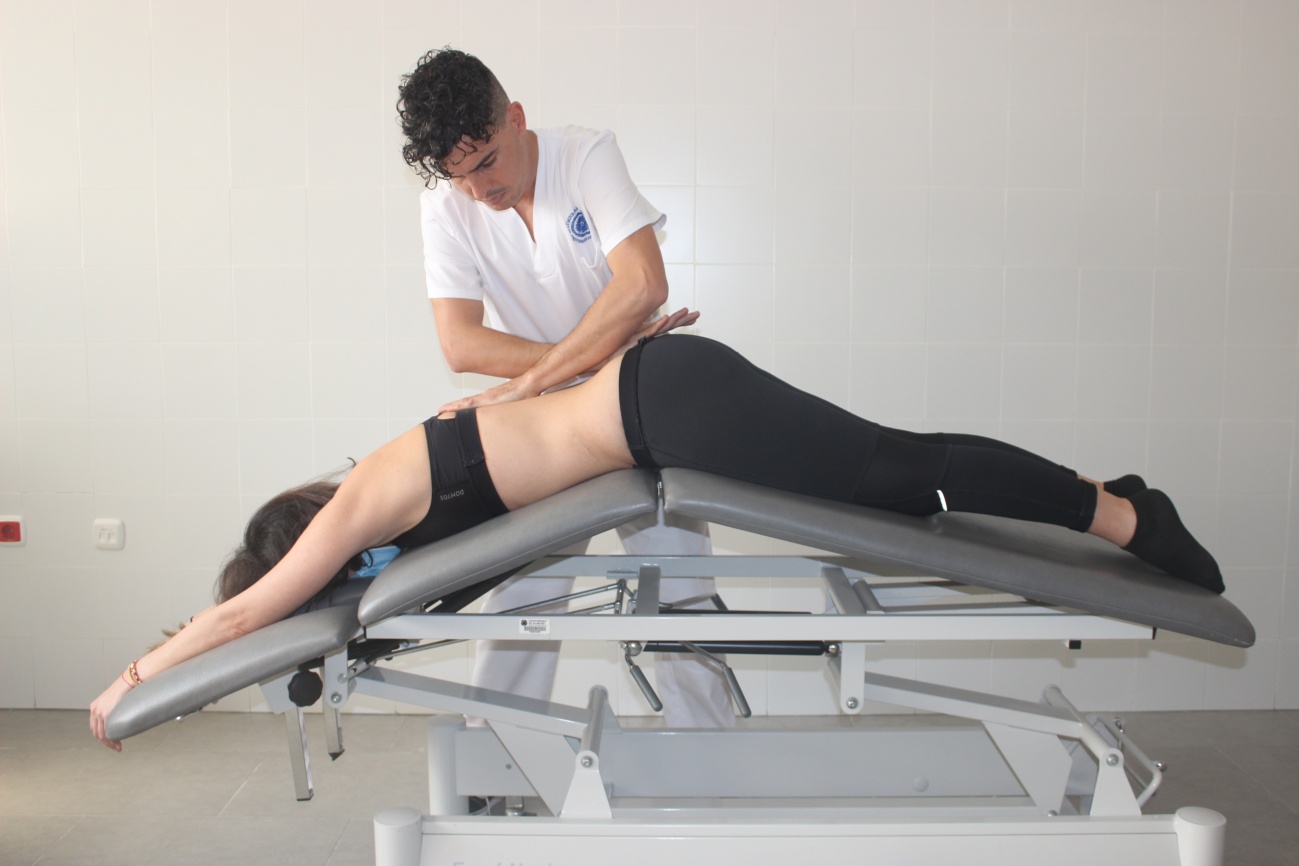


1. **Estiramiento del músculo multífido.**


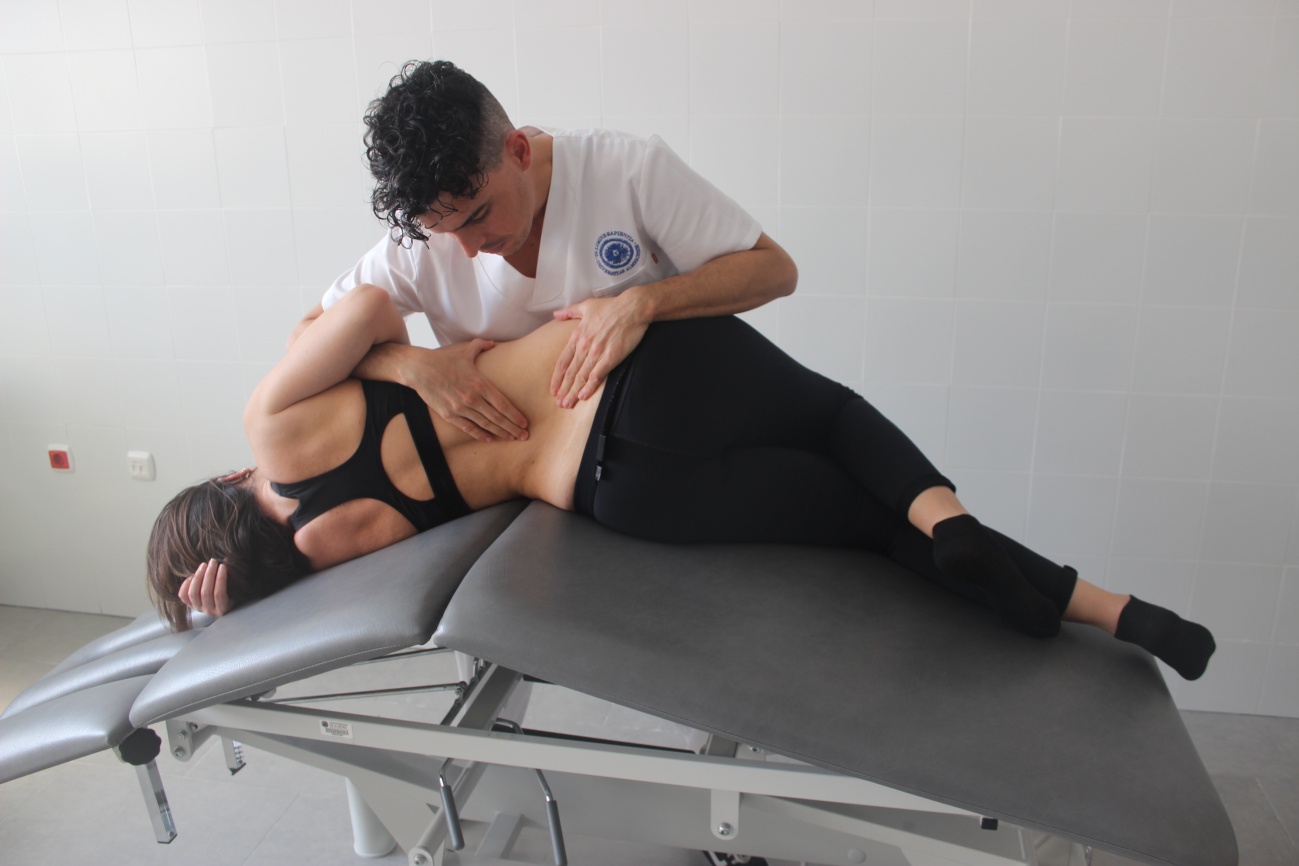


1. **Estiramiento del músculo cuadrado lumbar.**
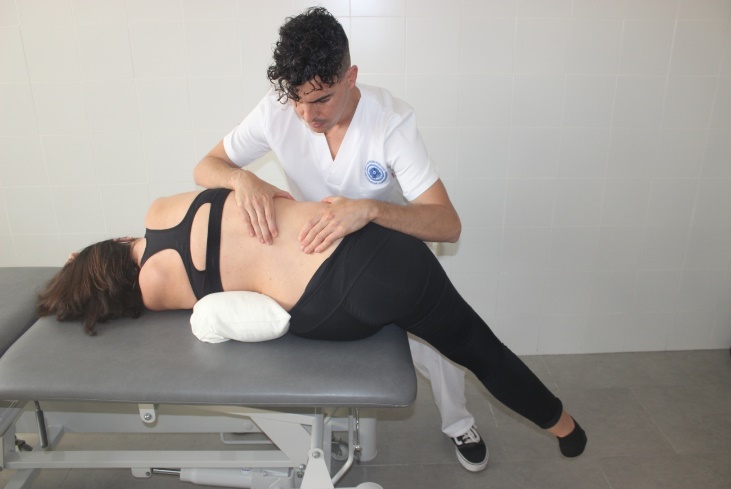

Supplement: Supplementary file 1 — Additional file 1:. Analytical stretching exercise protocol (lumbar segment). [file 13063_2022_6179_MOESM1_ESM.docx]
