# Supplementary material for: Electrical dry needling versus conventional physiotherapy in the treatment of active and latent myofascial trigger points in patients with nonspecific chronic low back pain
Source: Trials. 2022 Mar 28;23:238. doi: 10.1186/s13063-022-06179-y (PMC8961901; doi:10.1186/s13063-022-06179-y)

**APPENDIX 2. DOSSIER OF EXERCISES FOR LOW BACK PAIN (HOME PROGRAM)**

Starting Position: Lie supine (on your back) with your knees bent and both feet flat on the mat and the knees aligned with the second toe of each foot. Abduct your arms (extend to side) with the palms supinated (facing up to the ceiling).

**Exercise 1. Supine Pelvic Tilt**

Downward Phase: Exhale and gently contract your abdominal muscles to flatten your low back into the mat. Avoid trying to lift your hips (tailbone) off the mat. Hold this position briefly.

Upward Phase: Inhale and slowly relax your abdominal muscles while gently contracting your erector spinae (low back muscles) to increase the arch in your low back. Avoid raising your hips off the mat. Hold this position briefly before returning to your starting position.

Repeat 10 times


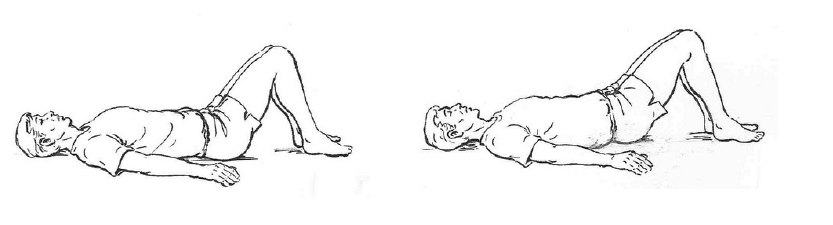


**Exercise 2.** Lower abs

Bend the neck and lower limbs, bringing the chin and knees to the chest. Hold for 5 seconds and return to the starting position.

Repeat 10 times


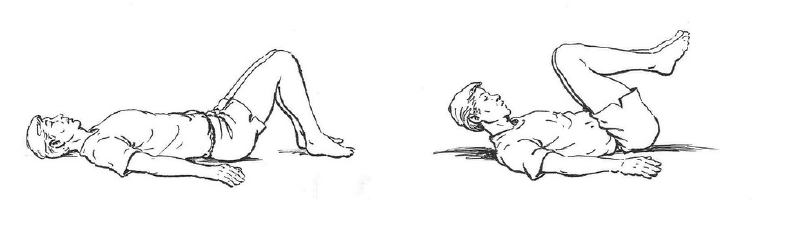


**Exercise 3. Upper abs**

Starting Position: Lie in a supine (on your back) position on a mat with your knees bent, feet flat on the floor and heels 12 - 18 "from your tailbone.

Bring the chin to the chest and raise the upper part of the trunk about 25 cm, touch the knees with the arms extended. Hold for 5 seconds and return to the starting position.


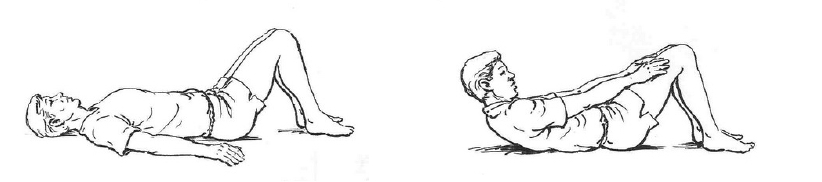
 Repeat 10 times

**Exercise 4. Obliques**

Sit on the floor with your knees bent. Hold your arms in front of your chest and lean back so your torso is at a 45-degree angle to the floor.

Without moving your torso, rotate your arms to the left as far as you can. Pause for 3 seconds before doing the same on the right.

Repeat 10 times


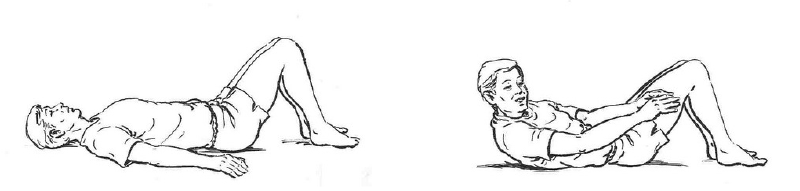


**Exercise 5. Glute Bridge**

Lying face up, bend the knees and press through the heels and raise the hips into the air by squeezing the glute muscles. Rest on the shoulders and the upper back while keeping the body in a straight line from the knees to the head. Hold for 5 seconds and lowly lower the hips back down to the ground.

Repeat 10 times


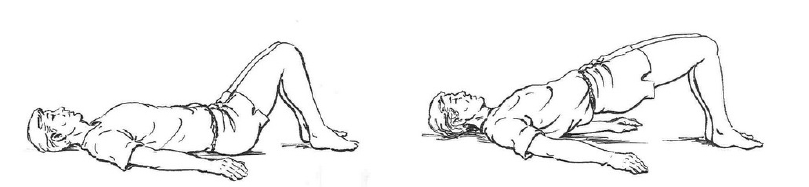


**Exercise 6. Prone Back Extension**

Lie on your stomach and your legs fully extended and your arms extended back. Engage your back muscles and lift your head and shoulders a little way upwards, bring your shoulder blades together gently; hold for a count of two, then slowly lower your trunk back to the starting position and relax completely. Hold for 5 seconds and return to the starting position.

Repeat 10 times


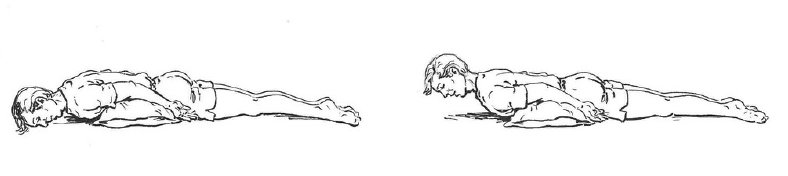


**Exercise 7. Extended leg raise**

Lie on your sid with your legs fully extended and stacked. Raise the upper leg 20-30 cm. Hold for 5 seconds and return to the starting position. Repeat with the other leg.

Repeat 10 times


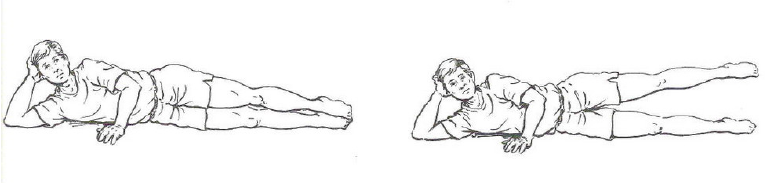


**Exercise 8. Prayer Stretch**


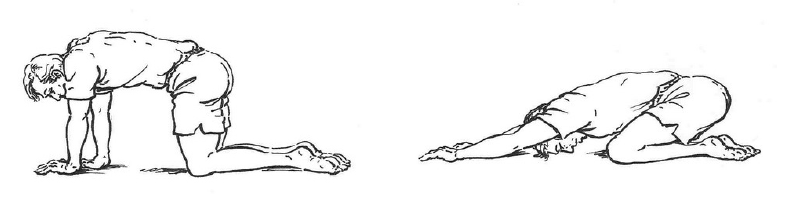
Bend your knees and hips until you sit on your heels, lean forward at the waist, extending your arms above your head until you reach the ground. flexing the neck at the same time. The goal is to lengthen the lumbar muscle. Hold for 30-60 seconds and sit back down. Repeat 3-5 times.

**Exercise 9. Cat-camel**

Start on all fours, positioning yours hands directly under yours shoulders and your knees under your hips. Drop your head and pull the abs in, rounding your back and neck. Hold for 10 seconds. Raise your head and curve your back down toward ther floor. Hold for another 10 seconds and repeat 5 times.


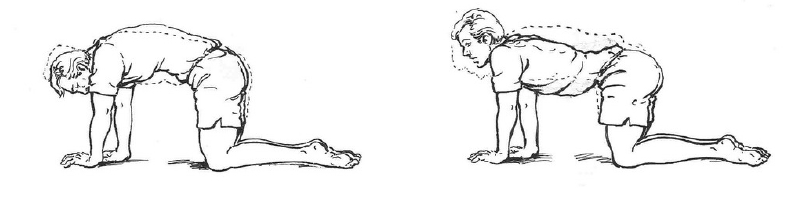


**Exercise 10. Quadruped Arm and Leg Raise**

Start on your hands and knees. Place your hands directly below your shoulders and align your head and neck so your spine creates a straight line. Tighten your abdominal muscles.

At the same time, straighten and extend your left arm and right leg, raising them until they are parallel to the floor. Hold for a breath, then lower using control. Repeat with your right arm and left leg.

Repeat 10 times


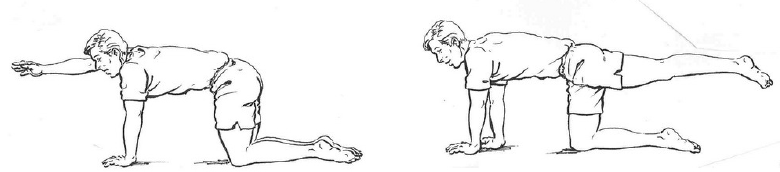

Supplement: Supplementary file 2 — Additional file 2:. Dossier of exercises for low back pain (home program). [file 13063_2022_6179_MOESM2_ESM.docx]
