# Supplementary material for: Electrical dry needling versus conventional physiotherapy in the treatment of active and latent myofascial trigger points in patients with nonspecific chronic low back pain
Source: Trials. 2022 Mar 28;23:238. doi: 10.1186/s13063-022-06179-y (PMC8961901; doi:10.1186/s13063-022-06179-y)
Supplement: Supplementary file 3 — Additional file 3:. Informed Consent. Information sheet for participants. [file 13063_2022_6179_MOESM3_ESM.docx]

**Appendix 3. Informed Consent. Information sheet for participants.**

MODEL CONSENT FORM–INFORMATION FOR PARTICIPANTS *(this document will be given to patients at the study inclusion visit, before baseline assessment and randomization is applied).*

Before signing this informed consent, carefully read the information provided below and ask the questions you consider appropriate in reference to the comparative study entitled: **“Electrical dry needling versus conventional physiotherapy in the treatment of active and latent myofascial trigger points in patients with nonspecific chronic low back pain”**

**Nature:**

♣ To compare the repercussions of electrical dry needling and conventional physical therapy in terms of pain, functionality, mobility of the spine and quality of life in patients with non-specific chronic low back pain.

♣ Analyze the repercussions on the active myofascial trigger points in terms of the pressure tolerance threshold after an intervention with Electrical dry needling and conventional physiotherapy.

**Importance:**

Many studies consulted conclude the importance of looking for alternatives in the forms of treatment that are effective in the symptomatology in people suffering from non-specific chronic low back pain due to the disability caused by this injury and the direct and indirect costs resulting from it, to achieve better results.

Our intention is to evaluate and compare the therapeutic approach using the electrical dry needling technique and conventional physiotherapy on the active myofascial trigger points of the lumbar spinal muscles in people diagnosed with chronic nonspecific low back pain.

**Study overview:**

Two treatment groups will be assigned randomly, G1 will receive dry electropuncture on active myofascial trigger points in the lumbar musculature, G2 will be treated by analytical stretching, inhibition of active myofascial trigger points and an exercise program at home to the lumbar muscles.

Treatment is aimed at the quadratus lumbar, multifidus and iliocostalis at the level of the lumbar spine. In the two groups, a total of 3 treatment sessions will be carried out, carrying out a weekly session. There will be an initial evaluation (pre-treatment), a final one after the last treatment session (post-treatment) and a short-term one (two months).

This evaluation will consist of: a series of tests and questionnaires that measure the degree of pain, mobility of the spine, functionality and quality of life together with the analysis of the mobility of the spine using Spinal Mouse technology and the pressure tolerance threshold with the Wagner pressure algometer clinical diagnostic tool.

• Participation is completely voluntary.

• The patient can withdraw from the study when they express it, without giving explanations and without this affecting their medical care.

• All personal data obtained in this study are confidential and will be treated in accordance with the Organic Law on the Protection of Personal Data 15/99.

• The information obtained will be used exclusively for the specific purposes of this study.

**Research risks to the patient:**

This research does not pose any risk to the participants.

If you require additional information, you can contact our research unit staff by phone: 686813652 or by email: hector-83@hotmail.com.

**INFORMED CONSENT - PARTICIPANT'S WRITTEN CONSENT**

**“Electrical dry needling versus conventional physiotherapy in the treatment of active and latent myofascial trigger points in patients with nonspecific chronic low back pain”**

I (Name and Surname): ........................................... ........................

• I have read the information document that accompanies this consent (Information to the Participant).

He podido hacer preguntas sobre el estudio: “Electrical dry needling versus conventional physiotherapy in the treatment of active and latent myofascial trigger points in patients with nonspecific chronic low back pain”

I have received enough information about the study: “Electrical dry needling versus conventional physiotherapy in the treatment of active and latent myofascial trigger points in patients with nonspecific chronic low back pain”

• I have spoken with the professional evaluator and informant: _____________________

• I understand that my participation is voluntary and I am free to participate or not in the study.

• I have been informed that all the data obtained in this study will be confidential and will be treated in accordance with the Organic Law on Protection of Personal Data 15/99.

• I have been informed that the information obtained will only be used for the specific purposes of the study.

**I understand that I can withdraw from the study:**

• Whenever you want.

• Without having to give explanations.

• Without this affecting my medical care.

• I freely give my consent to participate in the project entitled: “Electrical dry needling versus conventional physiotherapy in the treatment of active and latent myofascial trigger points in patients with nonspecific chronic low back pain”

Participant signature: Evaluator signature

Name and surname: Name and surname:

Date: Date:
